# New Experimental Spectra and Quantitative Modeling of the Bending Dyad of Silane in the 900 cm^−1^ Region for Its 3 Isotopologues

**DOI:** 10.3390/molecules30091992

**Published:** 2025-04-30

**Authors:** Raef Kamel, Vincent Boudon, Cyril Richard, Emna Raddaoui, Xavier Landsheere, Laurent Manceron, Fridolin Kwabia Tchana

**Affiliations:** 1 Laboratoire Interdisciplinaire Carnot de Bourgogne ICB, UMR 6303, Université Bourgogne Europe, CNRS, F-21000 Dijon, France; 2 CEA, DES, ISEC, DPME, Université de Montpellier, F-30200 Marcoule, France; 3 Université Paris Cité and Université Paris Est Créteil, CNRS, LISA, F-75013 Paris, France; 4 Synchrotron SOLEIL, Ligne AILES, L’Orme des Merisiers, St-Aubin BP48, 91192 Gif-sur-Yvette Cedex, France

**Keywords:** high-resolution molecular spectroscopy, silane, infrared

## Abstract

The silane (SiH_4_) molecule is of importance for a wide range of fundamental and applied problems of physics, chemistry, astrophysics, and industry. We present here the newest and clearest complete quantitative analysis and modeling of line positions and intensities in the strongly absorbing $\nu_{2}/\nu_{4}$ bending dyad in the 830–1150 cm^−1^ spectral region. A database of calculated silane lines, SiCaSDa, is made available online through the Virtual Atomic and Molecular Data Centre (VAMDC).

## 1. Introduction

More than nine decades have passed since the silane (SiH_4_) infrared absorption spectrum (IR) was originally recorded [1,2]. Subsequently, a number of high-resolution spectroscopic investigations have been conducted on SiH_4_ and its isotopologues, although these are now quite old. Notably, astronomical observations of rotation–vibration transitions have been made near the carbon star IRC + 10,216 [3]. Its presence is also suggested in the atmospheres of Jupiter [4] and Saturn [5], and SiH_4_ has already been considered in relation to biosignature gases on rocky exoplanets, although its detection seems quite unlikely [6]. This improbability arises from its thermodynamic instability in oxygen-rich environments, where it rapidly reacts with oxygen to form silicates or silicon dioxide, preventing its accumulation in significant amounts [7]. Furthermore, the biological production of SiH_4_ would require highly specialized and energy-intensive metabolic pathways, which have no known terrestrial analogs [8]. Nonetheless, its potential for abiotic production on certain planetary types has sustained interest in its study as a possible marker in exoplanetary research [6]. Silane gas finds extensive applications in the semiconductor manufacturing process and solar cell manufacturing industry.

The previous spectroscopic studies on this species were, however, performed quite a long time ago, and quantitative studies including the determination of absolution absorption intensities are quite sparse. The 900 cm^−1^ bending region was studied eight years ago by Ulenikov et al. using a similar experimental spectral resolution [9]. Our new study focuses primarily on analyzing the line positions and intensities with the application of Dijon’s tensorial formalism, aiming to improve the precision of the spectroscopic parameters and enhance the reliability of the generated line lists, in particular for absolute line intensities. As a matter of fact, modern applications to silane detection, silicon isotopic separation, etc., require a better and consistent reinvestigation of this spectroscopy in order to constitute reliable line lists.

This paper thus presents a comprehensive line position and intensity study of the silane bending region between 800 cm^−1^ and 1100 cm^−1^. Using new experimental data, we were able to fit an effective Hamiltonian and dipole moment operator for the $v_{2}=1$ and $v_{4}=1$ states by treating $\nu_{2}$ and $\nu_{4}$ as an interacting dyad. Experimentally, silane was used in natural abundance (92.22% ^28^SiH_4_, 4.68% ^29^SiH_4_, and 3.09% ^30^SiH_4_). In order to predict the absolute line intensities, we determined effective dipole moment parameter values for these two bands.

The experimental procedure is described in detail in Section 2, and the theoretical model created at Dijon is the subject of Section 3. The experimental line intensity measurements are shown in Section 4. Section 5 presents the results of effective Hamiltonian and dipole moment fits. Finally, Section 6 presents the SiCaSDa database of calculated silane absorption lines.

## 2. Experimental Details

**Figure 1:**
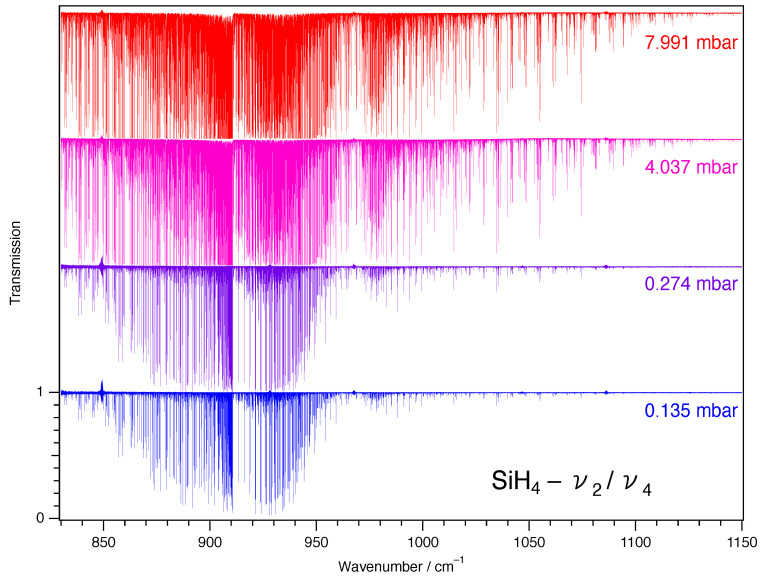
Overview spectra of the $\nu_{2}$/$\nu_{4}$ bands of silane in natural isotopic abundance, recorded at high resolution (0.0021 cm^−1^). The absorption path length was 5 cm, and the temperature was 300 K. In the current investigation, line intensities for that band were retrieved using the four spectra that were given (S2, S3, S7, and S8 in Table 1).

Eleven absorption spectra of silane (SiH_4_) have been recorded in the range 750 to 1300 cm^−1^ using the high-resolution Fourier transform spectrometer (FTS) Bruker IFS125HR located at the LISA facility in Créteil. The instrument was equipped with a silicon carbide Globar source, a KBr/Ge beamsplitter, and an optimized homemade MCT detector developed at the AILES beamline of the SOLEIL synchrotron facility [10], used in conjunction with a bandpass optical filter (810–1210 cm^−1^) to improve the signal-to-noise ratio (S/N). The FTS was continuously evacuated below 3 × 10^−4^ hPa by a turbomolecular pump to minimize absorption by atmospheric gases. The diameter of the entrance aperture of the spectrometer was set to 1.5 mm to maximize the intensity of IR radiation falling onto the detector without saturation or loss of spectral resolution. Interferograms were recorded with a 40 kHz scanner frequency and a maximum optical path difference (MOPD) of 428.57 cm. According to the Bruker definition (resolution = 0.9/MOPD), this corresponds to a resolution of 0.0021 cm^−1^. The spectra were obtained by Fourier transformation of the interferograms using a Mertz phase correction, 1 cm^−1^ phase resolution, a zero-filling factor of 2, and no apodization (boxcar option). Silane 5.0N (99.999% stated purity) was purchased from Linde Gas and used without further purification. For all the measurements, a small cryogenic cell (5.10 ± 0.01 cm path length) made of stainless steel to minimize adsorption and corrosion issues, fitted with 9.5 mm diameter, 0.4 mm thick wedged diamond windows (E6, The Netherlands) and housed inside the sample compartment of the Bruker IFS125HR spectrometer was used. A detailed description of the cell is given in Ref. [11]. The sample pressure in the cell was measured using calibrated MKS Baratron capacitance manometer models 627D (2 and 100 torr full scale) and 628D (10 torr full scale), characterized by a stated reading accuracy of 0.12%. Taking into account the uncertainty arising from small variations in the pressure during the recording (~0.35%), we estimated the measurement uncertainty on the pressure to be equal to 0.5%. All the spectra were recorded at a stabilized room temperature of 295 ± 1 K. The following procedure was used to record the spectra. A background spectrum was first collected while the cell was being continuously evacuated. It was recorded at the same resolution as the sample spectra to ensure proper removal of the weak channeling generated by the wedged cell windows. The infrared gas cell was then passivated several times with the SiH_4_ sample. Finally, spectra were recorded for eleven different sample pressures of silane. The eleven pressures chosen and the number of interferograms recorded and averaged to yield the corresponding spectra are listed in Table 1. All the sample spectra were ratioed against the empty cell background spectrum. The root mean square (RMS) S/N ratio in the ratioed spectra ranged between 550 and 930. For line position analysis, spectrum S2 was used and was calibrated by matching the measured positions of 39 lines of OCS observed in the 830–1100 cm^−1^ spectral region to reference wavenumbers available in HITRAN [11], with an RMS deviation of $4\times 10^{-5}$ cm^−1^. Figure 1 presents an overview of the $\nu_{2}$/$\nu_{4}$ region for a sample of silane in natural isotopic abundance. Four spectra were recorded and studied in the present work.

## 3. Theoretical Background

SiH_4_, just like CH_4_ [12], GeH_4_ [13], and SiF_4_ [14], has four normal modes of vibration [15]. It is a tetrahedral spherical top molecule with $T_{d}$ point group symmetry at equilibrium. Only the doubly degenerate mode with *E* symmetry ($\nu_{2}$) and the triply degenerate mode with $F_{2}$ symmetry ($\nu_{4}$) are studied in this work. The $\nu_{2}/\nu_{4}$ bending dyad area is formed by these two interacting bands, with respective centers approximately located at 970 cm^−1^ and 913 cm^−1^, respectively.

### 3.1. Effective Hamiltonian

The theoretical model used in this paper is based on the Dijon group’s tensorial formalism and vibrational extrapolation notion [16,17,18]. It makes complete use of the symmetry of the molecule. Let us quickly review the fundamental ideas of this model, which have already been covered in depth in Ref. [18], for instance. Here, we are considering an XH_4_ molecule, for which the ground state (GS) is represented by a set of polyads called $P_{k}$, where k=0,…,n and $P_{k}=0$ are the vibrational levels. Assuming that inter-polyad interactions have been eliminated through some perturbative treatment, such as a contact transformation [16], the Hamiltonian operator is expressed as follows:

(1) $$\begin{array}{c} H=H_{\left\{P_{0}\equiv \mathrm{GS}\right\}}+H_{\left\{P_{1}\right\}}+\ldots +H_{\left\{P_{k}\right\}}+\ldots +H_{\left\{P_{n-1}\right\}}+H_{\left\{P_{n}\right\}}, \end{array}$$

where the different $H_{\left\{P_{k}\right\}}$ terms are expressed in the following form:

(2) H{Pk}=∑allindexest{s}{s′}Ω(K,nΓ)ΓvΓv′β[V{s}{s′}Ωv(ΓvΓv′)Γε⊗RΩ(K,nΓ)](A1).

In this equation, the $t_{\left\{s\right\}\left\{s^{\prime }\right\}}^{\Omega \left(K,n\Gamma \right)\Gamma v\Gamma^{\prime }v}$ are the parameters to be determined, while V{s}{s′}Ωv(ΓvΓv′)Γε and $R^{\Omega (K,n\Gamma )}$ are vibrational and rotational operators, respectively. For each term, $\Omega_{v}$ and Ω represent the degree in elementary vibrational operators (creation $a^{+}$ and annihilation *a* operators) and rotational operators (components $J_{x}$, $J_{y}$, and $J_{z}$ of the angular momentum), respectively. $\varepsilon ={\left(-1\right)}^{\Omega }$ is the parity under time reversal. β is a factor that allows the scalar terms (terms with $\Gamma =A_{1}$, the totally symmetric irreducible representation of $T_{d}$) to match the “usual” contributions like $B_{0}J^{2}$, etc. The order of each individual term is defined as $\Omega +\Omega_{v}-2$. We deal with the effective Hamiltonians that are obtained for a given polyad $P_{k}$ by the projection of *H* on the $P_{n}$ Hilbert subspace:

(3) $$\begin{array}{ccc} {\tilde{H}}^{<P_{n}>} & = & P^{<P_{n}>}HP^{<P_{n}>}\\ & = & H_{\left\{\mathrm{GS}\right\}}^{<P_{n}>}+H_{\left\{P_{1}\right\}}^{<P_{n}>}+\ldots +H_{\left\{P_{k}\right\}}^{<P_{n}>}+\ldots +H_{\left\{P_{n-1}\right\}}^{<P_{n}>}+H_{\left\{P_{n}\right\}}^{<P_{n}>}. \end{array}$$

In the present case of silane, $\nu_{2}$ transitions are clearly visible, although this band is “forbidden” in absorption since it has an *E* symmetry. This, and the fact that $\nu_{2}$ and $\nu_{4}$ are close enough (≈60 cm^−1^), imply an intensity borrowing by Coriolis coupling. Since this is frequently the case with tetrahedral XY_4_ molecules, we will treat both bands as an interacting dyad [19,20,21].

We thus use the following effective Hamiltonians:

- The ground state effective Hamiltonian
  (4) $${\tilde{H}}^{<\mathrm{GS}>}=H_{\left\{\mathrm{GS}\right\}}^{<\mathrm{GS}>}.$$
- The $\nu_{2}/\nu_{4}$ bending dyad effective Hamiltonian
  (5) $${\tilde{H}}^{<\nu_{2}/\nu_{4}>}=H_{\left\{\mathrm{GS}\right\}}^{<\nu_{2}/\nu_{4}>}+H_{\left\{\nu_{2}/\nu_{4}\right\}}^{<\nu_{2}/\nu_{4}>}.$$

### 3.2. Effective Dipole Moment Operator

As our group recently conducted for instance regarding GeH_4_ [13], SiF_4_ [22], and RuO_4_ [23], we also need to expand the effective dipole moment operator in order to compute transition absolute intensities. As explained in Ref. [16], this operator is expanded as a sum of rotational–vibrational operators, much as the effective Hamiltonian. In the present case, it seems necessary to expand the $\nu_{2}/\nu_{4}$ bending dyad effective dipole moment up to order one plus one single second-order operator (with K=0 for the rotational contribution). This results in four operators in total, and four related parameters that need to be fitted with experimental line intensities:

(6) μ˜=μ21(R1(1,0F1)⊗V{GS}{ν2}1(A1E)E−)(F2)+μ40(R0(0,0A1)⊗V{GS}{ν4}1(A1F2)F2+)(F2)+μ41(R1(1,0F1)⊗V{GS}{ν4}1(A1F2)F2−)(F2)+μ42(R2(0,0A1)⊗V{GS}{ν4}1(A1F2)F2+)(F2),

where the effective parameters were represented using a simplified notation. The vibrational dipole moment derivative in this case is $\mu_{4}^{0}$ with respect to the $q_{4}$ normal mode coordinates, and the rotational–vibrational contribution $\mu_{4}^{1}$ corresponds to the standard Herman–Wallis factor [24]. The second-order rotational–vibrational correction is $\mu_{4}^{2}$, and the induced rotational–vibrational term for the $\nu_{2}$ band is $\mu_{2}^{1}$. This term is induced into the effective dipole moment operator by the contact transformation that isolates the bending dyad; this has been previously explained in the case of methane, CH_4_; see [19]. Similar to the effective Hamiltonian operator, the *R* and *V* symbols in Equation (6) stand for rotational and vibrational operators, respectively; their construction is described in Refs. [16,18]. Three further rotational–vibrational operators at order two (with K=2 for the rotational portion) exist in theory, but they cannot be determined here (see Section 5.2) and are therefore ignored.

### 3.3. Basis Sets

The coupled rotational–vibrational basis set is used to calculate the effective Hamiltonian and effective dipole moment matrix elements:

(7) $$|[\psi_{v}^{\left(C_{v}\right)}⊗\psi_{r}^{\left(J,nC_{r}\right)}{]}_{\sigma }^{\left(C\right)}\rangle ,$$

where $\psi_{r}^{\left(J,nC_{r}\right)}$ is a rotational wavefunction with angular momentum *J*, rotational symmetry species $C_{r}$, and multiplicity index *n*; $\psi_{v}^{\left(C_{v}\right)}$ is a coupled vibrational basis set; *C* is the overall symmetry species ($C=C_{v}⊗C_{r}$), with component σ. In the present case, $\psi_{v}^{\left(C_{v}\right)}$ contains the relevant functions for the $\nu_{2}$ and $\nu_{4}$ normal modes of vibration (bending modes)

(8) $$|\psi_{v\;\sigma_{v}}^{\left(C_{v}\right)}\rangle =|(\psi_{v_{2}}^{\left(l_{2},n_{2}C_{2}\right)}⊗\psi_{v_{3}}^{\left(l_{4},n_{4}C_{4}\right)}{)}_{\sigma_{v}}^{\left(C_{v}\right)}\rangle ,$$

with two contributions:

- For the doubly degenerate mode $\nu_{2}$, we use a symmetrized doubly degenerate harmonic oscillator basis set denoted
  (9) $$|\psi_{v_{2}\;\sigma_{2}}^{\left(l_{2},C_{2}\right)}\rangle =|v_{2},l_{2},C_{2},\sigma_{2}\rangle ,$$
  with $v_{2}=l_{2}=1$ for the dyad under consideration, $v_{2}$ and $l_{2}$ being the usual vibrational angular momentum quantum numbers and $C_{2}=E$.
- For the triply degenerate mode $\nu_{4}$, we use a symmetrized triply degenerate harmonic oscillator basis set denoted
  (10) $$|\psi_{v_{4}\;\sigma_{4}}^{\left(l_{4},n_{4}C_{4}\right)}\rangle =|v_{4},l_{4},n_{4},C_{4},\sigma_{4}\rangle ,$$
  with $v_{4}=l_{4}=1$ for the dyad under consideration, $v_{4}$ and $l_{4}$ being the usual vibrational angular momentum quantum numbers and $C_{4}=F_{2}$, while $n_{4}$ is a multiplicity index.

The effective Hamiltonian matrix is diagonalized numerically, and this leads to eigenfunctions obtained from

(11) $$\tilde{H}|\psi_{\sigma }^{\left(J,C,\alpha \right)}\rangle =E|\psi_{\sigma }^{\left(J,C,\alpha \right)}\rangle ,$$

where α=1,2,… number functions with the same symmetry *C* in a given *J* block. This eigenbasis set can be expanded in terms of the initial rotational–vibrational basis set (7) and is used to calculate the matrix elements (in Debye) of the *Z* component $\mu_{Z}$ of the effective dipole moment operator $\tilde{\mu }$ in the laboratory-fixed frame. This $\mu_{Z}$ component is related to $\tilde{\mu }$ from Equation (6) through Stone coefficients [25] and the direction cosines tensor, as explained in Ref. [18]. The line intensity at temperature *T* for a transition at wavenumber ${\tilde{\nu }}_{if}$ (in cm^−1^) between an initial state *i* (with energy $E_{i}$, in Joules) and a final state *f* is then obtained through

(12) $$S_{if}\left({\mathrm{cm}}^{-1}/\left(\mathrm{molecule}\;{\mathrm{cm}}^{-2}\right)\right)=\frac{1}{4\pi \varepsilon_{0}}{\tilde{\nu }}_{if}\frac{8\pi^{3}}{3hcQ}e^{-\frac{E_{i}}{k_{B}T}}\left(1-e^{-\frac{hc{\tilde{\nu }}_{if}}{k_{B}T}}\right)R_{if},$$

with

(13) $$R_{if}=3\sum_{\alpha_{i},\alpha_{f}}{\left|\langle \psi_{\sigma_{f}}^{\left(J_{i},C_{i},\alpha_{i}\right)}|\mu_{Z}|\psi_{\sigma_{f}}^{\left(J_{f},C_{f},\alpha_{f}\right)}\rangle \right|}^{2}.$$

*Q* is the total partition function at temperature *T*, *c* the speed of light in vacuum, *h* Planck’s constant, and $k_{B}$ Boltzmann’s constant. The line strength $S_{if}$ is expressed here in the so-called “HITRAN unit” [26,27].

## 4. Line Intensity Measurements and Uncertainty Analysis

### 4.1. Line Intensities

**Figure 2:**
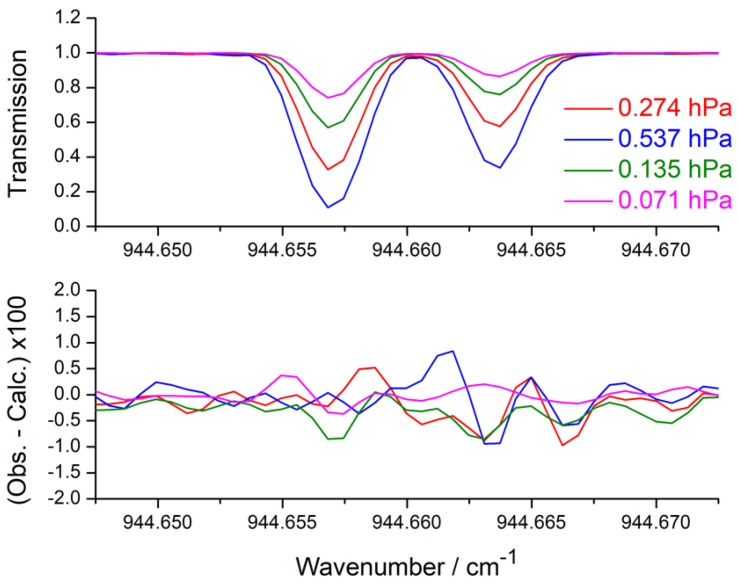
Results of the multi-spectrum analysis applied to four spectra (S1 to S4) of a small part of the $\nu_{4}$ band of SiH_4_. The top panel shows the observed spectra, and the lower panel presents the best-fit residuals corresponding to spectra S1 to S4.

The line intensities were measured using a multi-spectrum fitting program developed in Paris and previously used in several studies [28,29,30]. In this work, this program was applied to simultaneously fit 8 (for the strong $\nu_{4}$ band) or 5 (for the weaker $\nu_{2}$ band) experimental spectra of SiH_4_, constraining the fit of a transition by using the same set of line parameters for the calculation of this transition observed under various experimental conditions. Each synthetic spectrum is calculated as the convolution of the molecular transmission spectrum with an instrument line shape function, which includes the effects of the finite maximum optical path difference and the finite source aperture diameter of the interferometer. In the present work, no deviation from this model instrument line shape was observed using the nominal aperture diameter of 1.5 mm. The molecular transmission spectrum was generated on a wavenumber scale interpolated with respect to that of the corresponding observed spectrum. After convolution with the instrument line shape function, only the calculated spectral points corresponding to observed spectral points were retained. The background in each spectrum was represented by a polynomial expansion up to the second order (a constant or an affine function was, however, found sufficient in most cases), and the profile of the lines was modeled using a Voigt function with Gaussian width always held fixed to the value calculated for the Doppler broadening. The measurements were carried out on spectrally restricted intervals, ranging from 0.015 to 0.1 cm^−1^ and containing either a single or several lines. The fitted line parameters were the positions, intensities, and self-broadening coefficients. The self-broadening and self-shift coefficients of all the lines were set to 0.1 and 0.0 cm^−1^.atm^−1^, respectively. Figure 2 shows an example of this multi-spectrum analysis for a small part of the $\nu_{4}$ band.

The required initial values of the positions and intensities (as well as the assignments) of lines belonging to the $\nu_{4}$ and $\nu_{2}$ bands of the 3 isotopologues of silane considered in the present work were generated using rotational constants and transition dipole moment constants of SiH_4_ from a previous unpublished study included in the STDS package [31] of the XTDS software [32].

### 4.2. Uncertainty Analysis

Analysis of the fit residuals shows that they are generally less than 2%. However, to estimate the accuracy of the measured intensities also requires considering the uncertainties on the physical parameters, contributions from possible systematic errors [33], as well as the uncertainties derived from the fits, taken as the standard deviation. The various sources of error considered in the present work are sample impurity (0.001%), temperature (0.34%), pressure (0.5%), path length (0.2%), standard deviation from fit (<2%), and systematic errors. Analyses show that systematic errors and the standard deviations of the fits are the main sources of error. The dominant contributions to the systematic errors arise from shifts in the location of the full-scale (100%) photometric level or measurement noise. The effect of noise and small baseline offsets in these were also considered. The 0% level could be checked readily and the 100% level fitted on the spectra in regions without absorptions. In the end, for systematic errors, an arbitrary but conservative estimate of 2% of the line intensities has been retained here. For each transition, we then calculated an upper limit for the overall uncertainty based on the maximum uncertainty of the individual experimental parameters, i.e., $\varepsilon_{\mathrm{si}}$ (sample purity), $\varepsilon_{t}$ (temperature), $\varepsilon_{p}$ (pressure), $\varepsilon_{\mathrm{pl}}$ (pathlength), $\varepsilon_{\mathrm{fit}}$ (standard deviation from fit), and $\varepsilon_{\mathrm{sys}}$ (systematic errors), assuming that these uncertainties are uncorrelated:

(14) $$\varepsilon =\sqrt{\varepsilon_{\mathrm{si}}^{2}+\varepsilon_{t}^{2}+\varepsilon_{p}^{2}+\varepsilon_{\mathrm{pl}}^{2}+\varepsilon_{\mathrm{fit}}^{2}+\varepsilon_{\mathrm{sys}}^{2}}$$

Using Equation (14) and the experimental relative uncertainties involved, the estimated overall uncertainty (estimated accuracy) for each of the 1024 line intensities measured was calculated. On average, the estimated accuracy for line intensities is equal to 5%.

## 5. Analysis and Discussion

### 5.1. Line Positions

Only experimental spectra S2 and S8 were used in the line position analysis of the $\nu_{2}/\nu_{4}$ bending dyad, as shown in Table 1. Due to the natural abundance of three silane isotopologues, these spectra have a dense structure with overlapping transitions. Using the parameter results from the STDS (spherical-top data system) package [31], which is a component of the XTDS (extended spherical-top data system) program [32], as the initial values, we began the analysis of the most prevalent isotopologue, ^28^SiH_4_.

The calculations using these parameters resulted in a highly accurate initial spectrum simulation, enabling the assignment of numerous transitions in the *P* and *R* branches. These assignments were carried out with the homemade software SPVIEW (Spectrum-View) [32], in its latest 2.0.2 version (https://icb.u-bourgogne.fr/spview, accessed on 1 June 2024). In contrast, the *Q* branch exhibited a higher density of transitions, making assignments more challenging. Nevertheless, by employing a standard iterative Levenberg–Marquardt non-linear least-squares fitting procedure, alongside simulations and new assignment sets, a total of 2464 transitions of ^28^SiH_4_ were successfully assigned, reaching J=25. This was achieved using a set of 29 parameters, with a root-mean-square deviation of 3.53 × 10^−4^ cm^−1^, as summarized in Table 2. Each line position was assumed to have an uncertainty of 1 × 10^−3^ cm^−1^, accounting for both line position and global calibration errors. This value was chosen as a conservative estimate based on the resolution of our spectrometer, the quality of the S/N ratio, and the peak-by-peak fitting procedure used in SPVIEW to extract line positions (assuming Voigt profiles). Table 3 represents the 10 ground state parameters that were fixed to their fitted values as reported in Ref. [34]. We consider here that these ground state parameters do not depend on the isotope of the central atom.

After completing the transition assignments for the primary isotopologue, the same parameters were applied to analyze the less abundant isotopologues. For these, the band center was first adjusted to visually align most transitions, and the same assignment fitting procedure was then repeated. Parameters that could not be reliably fitted were fixed to the values determined for Ref. [34].

The results for all three isotopologues are summarized in four tables. Table 4 describes nine parameters for the $v_{2}=1$ vibrational level up to the fifth order. Table 5 defines seventeen parameters for the $v_{4}=1$ vibrational level up to the fifth order too, and Table 6 shows the three interaction parameters accounting for couplings between $v_{2}=1$ and $v_{4}=1$ states. The last column of the table provides a conversion of all the constant values into the “classical” notation. The fit achieved excellent accuracy, with root-mean-square deviations of 3.44 × 10^−4^ cm^−1^, 2.72 × 10^−4^ cm^−1^, and 7.67 × 10^−4^ cm^−1^ for each isotopologue (^28^SiH_4_, ^29^SiH_4_, and ^30^SiH_4_), respectively. All the fit statistics are shown in Table 2.

Table 3, Table 4 and Table 5 also provide a comparison with the previous results of Ulenikov et al. [9] and show not primarily differences but more precision. The detailed comparison parameter by parameter is not always obvious to perform since we did not always fit the same parameters (technically, this corresponds to a different reduction choice).

**Figure 3:**
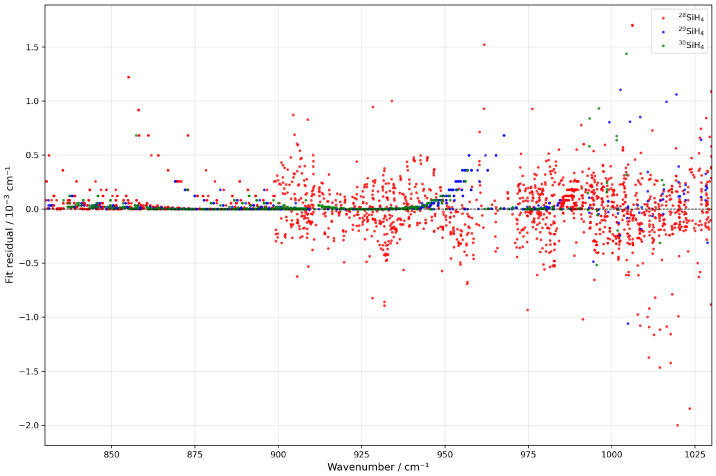
Fit residuals for line positions for the three isotopologues under consideration as a function of the wavenumber.

Figure 3 illustrates the residuals from the line position fit. A slight polynomial deviation is noticeable in the $\nu_{4}$ region below 925 cm^−1^ for $\nu_{2}$ in the region above 940 cm^−1^, potentially indicating higher-order contributions to the effective Hamiltonian that were not accounted for in this fit. Nevertheless, the residuals remain minimal overall.

**Figure 4:**
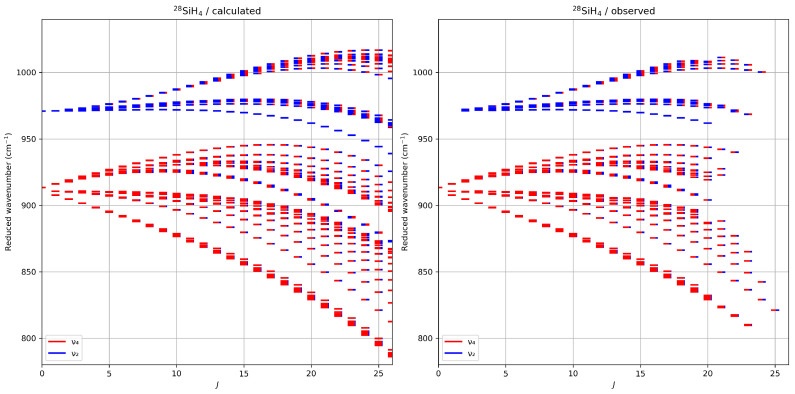
Calculated (**left panel**) and observed (**right panel**) reduced energy levels for ^28^SiH_4_, as a function of the rotational quantum number *J*. Observed levels correspond to levels reached by assigned transitions. The colors indicate the mixings between both vibrational levels, i.e., the projection of each eigenlevel onto the $v_{2}=1$ (red) and $v_{4}=1$ (blue) initial normal mode basis sets.

Figure 4 presents the reduced energy levels for ^28^SiH_4_, calculated as

(15) $$\begin{array}{ccc} {\tilde{\nu }}_{\mathrm{red}} & = & \tilde{\nu }-\sum_{\Omega }t_{\left\{\mathrm{GS}\}\{\mathrm{GS}\right\}}^{\Omega \left(0,0A_{1}\right)A_{1}A_{1}}{\left(J\left(J+1\right)\right)}^{\Omega /2}\\ & = & \tilde{\nu }-B_{0}J\left(J+1\right)+D_{0}J^{2}{\left(J+1\right)}^{2}-\ldots , \end{array}$$

where $B_{0}$, $D_{0}$, and subsequent terms represent ground-state values. This formulation subtracts the dominant scalar polynomial terms, thereby highlighting level splittings arising from molecular symmetry.

The colors in the figure illustrate the mixing of energy levels caused by interactions between the two vibrational states. Both calculated and observed reduced energy levels are displayed. The observed levels correspond to those accessed by assigned transitions included in the fit, providing insight into the sampled energy spectrum. A similar pattern is observed for the two other isotopologues.

This figure serves as a valuable tool for assessing the accuracy of simulations based on the current effective Hamiltonian parameters, particularly when extrapolating to unassigned *J*-values. A detailed examination of the isotopic dependence of band centers and Coriolis interaction parameters, using a simplified model and the present values, can be found in Ref. [36].

### 5.2. Line Intensities

As detailed in Section 4, the predictions of line positions and relative intensities obtained from the effective Hamiltonian fit presented in Section 5.1 were essential for the extraction of experimental absolute line intensities. By comparing these theoretical predictions with experimental line positions, we were able to assign the corresponding lines for an intensity fit. Subsequently, for each isotopologue, we performed a fit of the four effective dipole moment parameters up to second order, as outlined in Section 3.2 (note that higher-order operators are not included in this fit as they cannot be determined and are excluded from the analysis). In this fitting procedure, only lines with a relative observed-minus-calculated difference of less than 10% were considered. This approach enabled an extended intensity analysis of the bending dyad of silane, using several hundred intensity data points for each isotopologue.

The effective dipole moment parameters and the associated fit statistics are summarized in Table 7. The results, using more than 500 data (measured line intensities), indicate excellent agreement, with a relative standard deviation below 4.1% for all the isotopologues. Compared with the oldest results in Ref. [9], the difference is not huge: we have almost the same result but with more parameters of higher order and a *d*_RMS_ almost two times better for the most abundant isotope ^28^SiH_4_.

**Figure 5:**
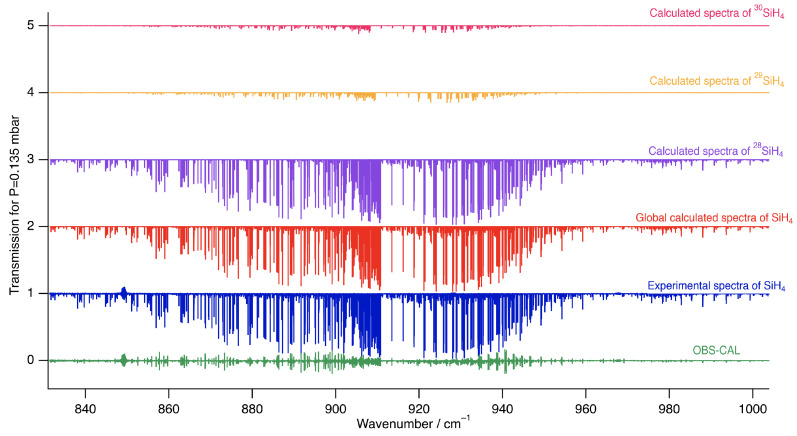
An overview of the spectrum simulations for all three silane isotopologues in their natural abundance. The red curve represents the total simulation, incorporating all isotopologues. The experimental spectrum, shown in blue, using spectrum S2 for the $\nu_{4}$ and $\nu_{2}$ bands, recorded under different conditions to obtain residual simulations for both positions detailed in Table 6 and intensities detailed in Table 7.

Figure 5 presents a simulation of the spectra for the three silane isotopologues in their natural abundance based on the parameters obtained from the fits of line positions and intensities described earlier.

**Figure 6:**
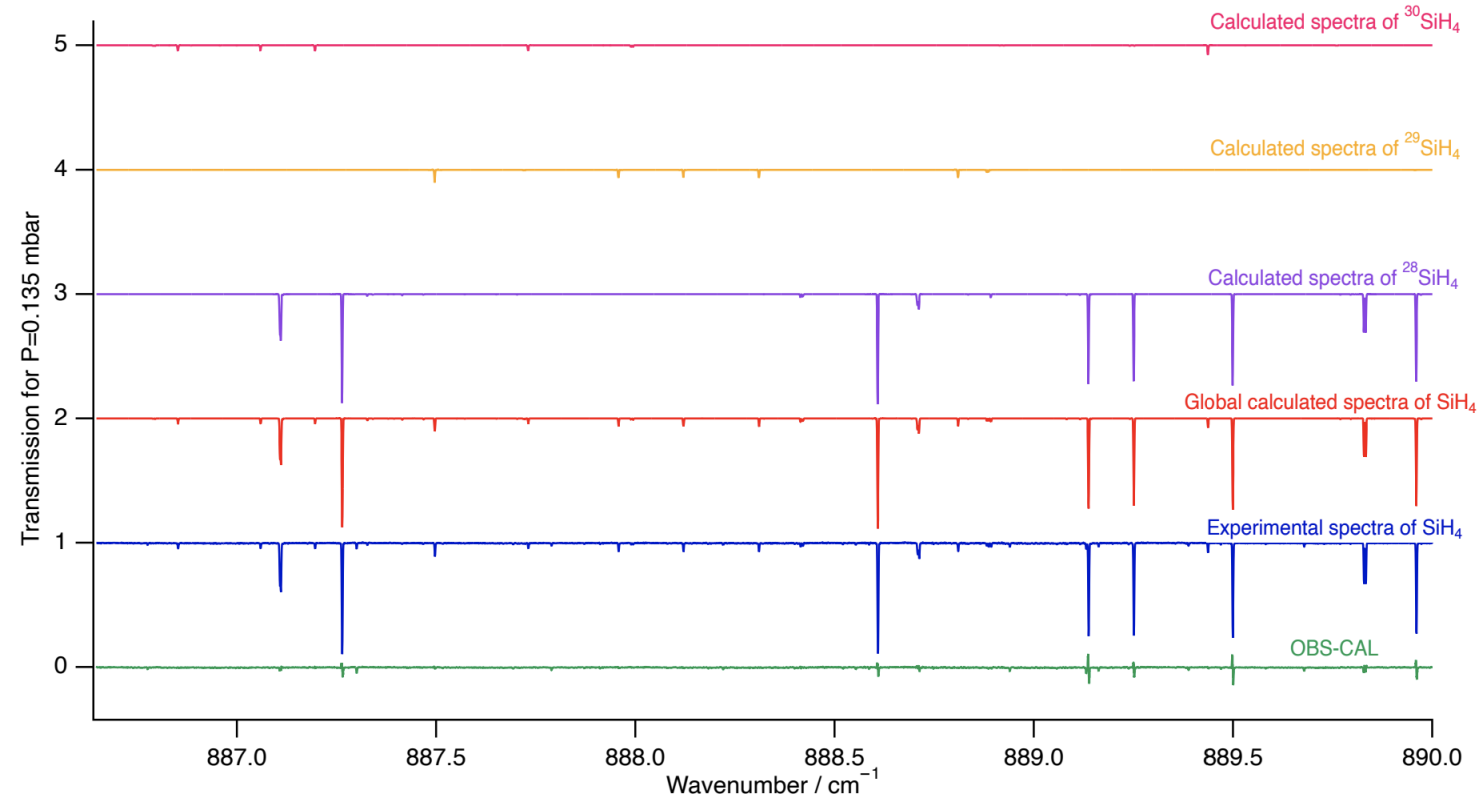
Zoomed-in simulation of the $\nu_{2}/\nu_{4}$ stretching dyad in the P-branch region compared to experimental data.

**Figure 7:**
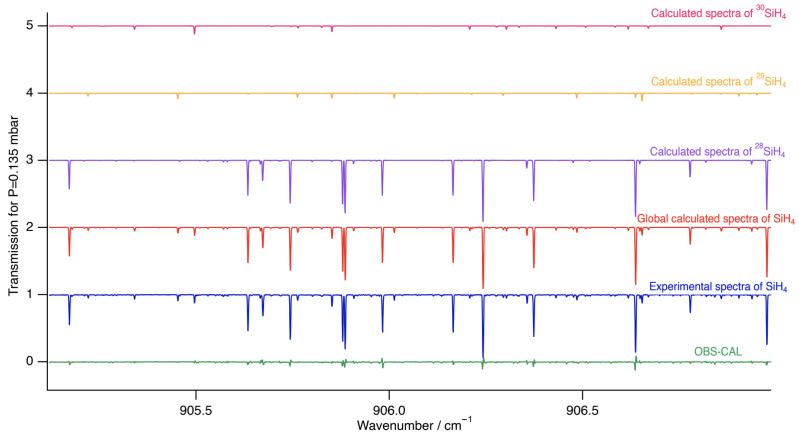
Zoomed-in simulation of the $\nu_{4}$ stretching dyad in the Q-branch region compared to experimental data.

Figure 6 and Figure 7 show zoomed-in perspectives of the $\nu_{4}$ *P* and *Q* branches, respectively, to observe the comparison and the difference between the experimental and simulated spectra more closely.

## 6. SiCaSDa Database Update

**Figure 8:**
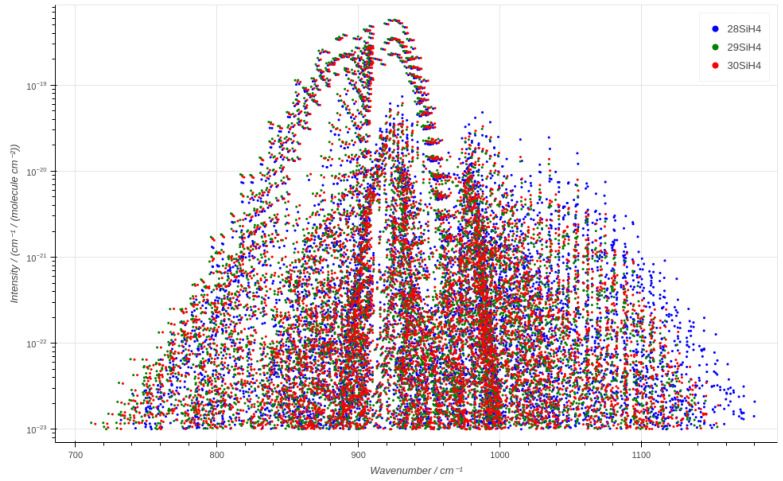
Interactive output from SiCaSDa between 700 and 1200 cm^−1^ for the three isotopologues.

As part of the CaSDa24 [37] project, we have set up the SiCaSDa database by using a polyad scheme adapted to the $\nu_{2}/\nu_{4}$ bending dyad. The data are available at the following address: https://vamdc.icb.cnrs.fr/PHP/SiH4.php either as a line list in HITRAN2004 format [38] or as cross-section data, which are calculated by summing binned intensities, in a 2-column flat file. Querying in the 700 and 1200 cm^−1^ range, which corresponds to the analysis performed in this paper, returns an interactive plot, as shown in Figure 8. Table 8 illustrates the 20,090 lines that were integrated, together with the pure rotational data already available (13,820 lines) in SiCaSDa.

## 7. Conclusions and Perspectives

In this study, we analyzed several absorption spectra of silane in the region near 900 cm^−1^ where two fundamental bands forming the bending dyad $\nu_{2}/\nu_{4}$ are located. The analysis focused mainly on both the line positions and line intensities of the three isotopologues of SiH_4_ in natural abundance (92.22% ^28^SiH_4_, 4.68% ^29^SiH_4_, and 3.09% ^30^SiH_4_) using more than 3500 transitions for the line position with an average root mean square $d_{\mathrm{RMS}}=3.6\%$ and with a $J_{\max}=25$ for ^28^SiH_4_. Rotational, centrifugal distortion, tetrahedral splitting, and interaction parameters for the ground (0100) and (0001) vibrational states were derived from the fitting of experimental line positions. The resulting parameters accurately reproduce the original experimental data, with deviations comparable to the experimental uncertainties. Approximately 550 experimental rotational–vibrational lines of ^28^SiH_4_ in the dyad region were analyzed using Voigt profiles to model the observed line shapes and extract experimental line intensities. Based on these results, a set of seven effective dipole moment parameters for the dyad of ^28^SiH_4_ were determined through a weighted fit, achieving a deviation of root-mean-square intensity errors $d_{\mathrm{RMS}}=3.2\%$. In order to conduct a full analysis of the other fundamental and combination bands, it is now necessary to study other vibrational states.

## Figures and Tables

**Table 1:** Pressure (*P*) of SiH_4_ (in hPa) and number of interferograms averaged to yield the corresponding spectrum (#scans). All the spectra were recorded with an absorption path length of 5.10 ± 0.01 cm at a stabilized room temperature of 295 ± 1 K, a resolution (equal to 0.9 divided by the maximum optical path difference) 0.0021 cm^−1^, and an entrance aperture diameter of the interferometer equal to 1.5 mm. The absolute uncertainty on the pressure is equal to 0.5% of the value given.

Spectrum	*P* (SiH_4_)/hPa	#Scans
S1	0.0710 (4)	396
S2	0.1350 (8)	364
S3	0.2740 (14)	210
S4	0.5370 (27)	206
S5	1.0040 (50)	392
S6	2.014 (10)	416
S7	4.037 (20)	404
S8	7.991 (40)	500
S9	16.02 (8)	358
S10	32.00 (16)	412
S11	63.95 (32)	404

**Table 2:** Fit statistics of all three isotopologues.

Isotope	Abundance	Nb. Data	$J_{\max}$	$d_{\mathrm{RMS}}$ *
^28^SiH_4_	92.23	2464	25	0.353
^29^SiH_4_	4.67	592	22	0.275
^30^SiH_4_	3.10	514	24	0.769

* $d_{\mathrm{RMS}}/10^{-3}$ cm^−1^.

**Table 3:** Effective Hamiltonian parameters for the ground vibrational states (GSs) of all three isotopologues of silane (SiH_4_). Standard deviation is indicated in parentheses in the unit of the last two digits.

Order	Ω(K,nC)	$\left\{s\right\}\;C_{1}$	$\left\{s^{\prime }\right\}\;C_{2}$	Value/cm^−1^		$f^{\;‡}$	Notation of Robiette et al. [35]
				^28^SiH_4_ Ref. [9]	Ref. [34]		
0	$2\;(0,0A_{1})$	$0000A_{1}$	$0000A_{1}$	2.85904477(47)	2.8590790619 ^†^		$B_{0}$
2	$4\;(0,0A_{1})$	$0000A_{1}$	$0000A_{1}$	−3.66810(33)	−3.6917820769 ^†^	×10^−5^	$-D_{0}$
2	$4\;(4,0A_{1})$	$0000A_{1}$	$0000A_{1}$	−1.707350(99)	−1.7071029198 ^†^	×10 ^−6^	$-\left(\sqrt{15}/4\sqrt{2}\right)D_{0t}$
4	$6\;(0,0A_{1})$	$0000A_{1}$	$0000A_{1}$	1.3495(93)	1.9961361842 ^†^	×10^−9^	$H_{0}$
4	$6\;(4,0A_{1})$	$0000A_{1}$	$0000A_{1}$	−6.034(27)	−5.9757956144 ^†^	×10^−11^	$\left(3\sqrt{5}/16\sqrt{2}\right)H_{4t}$
4	$6\;(6,0A_{1})$	$0000A_{1}$	$0000A_{1}$	−1.4730(75)	−1.4561902784 ^†^	×10^−11^	$-\left(\sqrt{231}/64\sqrt{2}\right)H_{4t}$
5	$8\;(0,0A_{1})$	$0000A_{1}$	$0000A_{1}$	−9.59(88)	−6.0184004392 ^†^	×10^−13^	$L_{0}$
5	$8\;(4,0A_{1})$	$0000A_{1}$	$0000A_{1}$	−2.17(17)	−1.9811867799 ^†^	×10^−15^	$\left(3\sqrt{15}/64\sqrt{2}\right)L_{4t}$
5	$8\;(6,0A_{1})$	$0000A_{1}$	$0000A_{1}$	−10.39(81)	−9.2968388902 ^†^	×10^−16^	$-\left(3\sqrt{77}/256\sqrt{2}\right)L_{6t}$
5	$8\;(8,0A_{1})$	$0000A_{1}$	$0000A_{1}$		−1.4443328483 ^†^	×10^−16^	$-\left(1/32\sqrt{33}\right)L_{8t}$

^†^ Ground state parameters are fixed to ^28^SiH_4_ values. ^‡^ This factor applies to the two previous columns.

**Table 4:** Effective Hamiltonian parameters for the $v_{2}=1$ vibrational level of all three isotopologues of silane (SiH_4_). Standard deviation is indicated in parentheses in the unit of last two digits.

Order	Ω(K,nC)	$\left\{s\right\}\;C_{1}$	$\left\{s^{\prime }\right\}\;C_{2}$			Value/cm^−1^				$f^{\;‡}$	Notation of Robiette et al. [35]
				^28^SiH_4_ Ref. [9]	^28^SiH_4_	^29^SiH_4_ Ref. [9]	^29^SiH_4_	^30^SiH_4_ Ref. [9]	^30^SiH_4_		(When Defined)
0	$0\;(0,0A_{1})$	0100E	0100E	970.9344501 (89)	970.93468 (13)	970.9484153 (47)	970.94853 (37)	970.9614826 (76)	970.96186 (36)		$\nu_{2}$
2	$2\;(0,0A_{1})$	0100E	0100E		−3.326 (25)	−0.05107	−4.17 (47)	−0.10214 (75)	−1.24 (36)	×10^−3^	$B_{2}-B_{0}$
2	2(2,0E)	0100E	0100E	−0.965273 (30)	−1.2545 (22)	−0.969766	−1.330 (42)	0.974259 (69)	−1.074 (31)	×10^−2^	$\sqrt{3}b_{2}+24\left(\sqrt{3}/7\right)C_{6}$
3	$3\;(3,0A_{2})$	0100E	0100E	2.4228 (54)	3.052 (23)	†	3.63 (42)	†	2.75 (11)	×10^−5^	$\left(1/2\right)d_{2}$
4	$4\;(0,0A_{1})$	0100E	0100E		−8.07 (12)		−7.01 (87)		−5.44 (82)	×10^−7^	$-\left(D_{2}-D_{0}\right)$
4	4(2,0E)	0100E	0100E	−3.927 (22)	−5.49 (61)	†	−5.3 (3.5)	†	−3.27 (50)	×10^−8^	$-\left(3/4\right)C_{5}+\left(9/7\right)C_{6}$
4	$4\;(4,0A_{1})$	0100E	0100E		−2.826 (40)		−2.77 (26)		−5.8 (1.7)	×10^−7^	$-\left(\sqrt{15}/4\right)\left(D_{2t}-D_{1t}\right)$
4	4(4,0E)	0100E	0100E		4.434 (62)		3.83 (78)		3.55 (18)	×10^−7^	$-\left(3\sqrt{3/7}\right)C_{6}$
5	$5\;(3,0A_{2})$	0100E	0100E	−0.17611 (81)	−3.04 (88)	†	†	†	†	×10^−10^	

^†^ Fixed value. ^‡^ This factor applies to the six previous columns.

**Table 5:** Effective Hamiltonian parameters for the $v_{4}=1$ vibrational level of all three isotopologues of silane (SiH_4_). Standard deviation is indicated in parentheses in the unit of the last two digits.

Order	Ω(K,nC)	$\left\{s\right\}\;C_{1}$	$\left\{s^{\prime }\right\}\;C_{2}$			Value/cm^−1^				$f^{\;‡}$	Notation of Robiette et al. [35]
				^28^SiH_4_ Ref. [9]	^28^SiH_4_	^29^SiH_4_ Ref. [9]	^29^SiH_4_	^30^SiH_4_ Ref. [9]	^30^SiH_4_		(When Defined)
0	$0\;(0,0A_{1})$	$0001F_{2}$	$0001F_{2}$	913.4687855 (78)	913.46833 (11)		912.18249 (19)		910.97900 (17)		$\nu_{4}$
1	$1\;(1,0F_{1})$	$0001F_{2}$	$0001F_{2}$	6.0271851 (21)	6.030710 (42)	6.0520511 (16)	6.05634 (49)	6.0752223 (19)	6.07642 (37)		$3\sqrt{2}$$B\zeta_{4}$$(\nu_{4}$ Coriolis)
2	$2\;(0,0A_{1})$	$0001F_{2}$	$0001F_{2}$	3.9654 (16)	2.610 (16)	4.04084 (36)	3.15 (31)	4.1519 (50)	1.17 (24)	×10^−3^	$B_{4}-B_{0}$
2	2(2,0E)	$0001F_{2}$	$0001F_{2}$	−3.05434 (18)	−6.373 (24)	−3.061987 (68)	−7.17 (47)	−3.07064 (80)	−4.20 (35)	×10^−3^	$-\left(1/2\right)\alpha_{220}-6\alpha_{224}$
2	$2\;(2,0F_{2})$	$0001F_{2}$	$0001F_{2}$	−12.22340 (24)	−9.716 (19)	−12.17770 (60)	−9.06 (36)	−12.13367 (55)	−10.128 (27)	×10^−3^	$-\left(3/4\right)\alpha_{220}+6\alpha_{224}$
3	$3\;(1,0F_{1})$	$0001F_{2}$	$0001F_{2}$	6.8348 (88)	5.718 (33)	6.8677 (36)	4.96 (60)	6.8851 (47)	6.44 (18)	×10^−5^	$-(3\left(\sqrt{3}\right)/4\left(\sqrt{2}\right)F_{110}$
3	$3\;(3,0F_{1})$	$0001F_{2}$	$0001F_{2}$	−5.3703 (69)	−6.306 (32)	†	−7.04 (58)	†	−5.69 (17)	×10^−5^	$\left(3/\sqrt{5}/2\right)F_{134}$
4	$4\;(0,0A_{1})$	$0001F_{2}$	$0001F_{2}$	−7.0296 (83)	−1.373 (85)	†	−1.99 (61)	†	−3.04 (54)	×10^−7^	$-\left(D_{4}-D_{0}\right)$
4	4(2,0E)	$0001F_{2}$	$0001F_{2}$	2.556 (17)	2.749 (96)	†	1.91 (82)	†	−8.6 (3.6)	×10^−7^	$\left(\sqrt{3}/8\right)G_{220}+\left(3\sqrt{3}/2\right)G_{224}$
4	$4\;(2,0F_{2})$	$0001F_{2}$	$0001F_{2}$	2.785 (69)	2.260 (43)	†	2.13 (54)	†	2.44 (26)	×10^−7^	
4	$4\;(4,0A_{1})$	$0001F_{2}$	$0001F_{2}$	2.19 (10)	1.871 (32)	†	1.68 (34)	†	5.0 (1.3)	×10^−7^	$\left(3\sqrt{3}/16\right)G_{220}-\left(3\sqrt{3}/2\right)G_{224}$
4	4(4,0E)	$0001F_{2}$	$0001F_{2}$	2.808 (14)	5.488 (47)	†	6.17 (40)	†	4.30 (40)	×10^−7^	$\left(-3\sqrt{5}/4\sqrt{2}\right)\left(D_{4t}-D_{0t}\right)$
4	$4\;(4,0F_{2})$	$0001F_{2}$	$0001F_{2}$	2.028 (20)	5.679 (35)	†	5.44 (39)	†	6.78 (21)	×10^−7^	
5	$5\;(1,0F_{1})$	$0001F_{2}$	$0001F_{2}$		2.93 (12)		2.36 (65)		3.43 (65)	×10^−9^	$\left(-3\sqrt{7}/2\right)G_{244}+\left(\sqrt{21}/2\sqrt{22}\right)G_{246}$
5	$5\;(3,0F_{1})$	$0001F_{2}$	$0001F_{2}$	−3.176 (19)	1.08 (12)	†	1.73 (60)	†	†	×10^−9^	$\left(-9\sqrt{7}/8\right)G_{244}-\left(\sqrt{21}/\sqrt{22}\right)G_{246}$
5	$5\;(5,0F_{1})$	$0001F_{2}$	$0001F_{2}$		3.225 (61)		3.21 (36)		1.03 (31)	×10^−9^	
5	$5\;(5,1F_{1})$	$0001F_{2}$	$0001F_{2}$	5.71 (19)	4.33 (86)	†	†	†	†	×10^−10^	

^†^ Fixed value. ^‡^ This factor applies to the six previous columns.

**Table 6:** Effective Hamiltonian parameters for the $v_{2}=1$ and $v_{4}=1$ interaction levels of all three isotopologues of silane (SiH_4_). Standard deviation is indicated in parentheses in the unit of last two digits.

Order	Ω(K,nC)	$\left\{s\right\}\;C_{1}$	$\left\{s^{\prime }\right\}\;C_{2}$			Value/cm^−1^				$f^{\;‡}$	Notation of Robiette et al. [35]
				^28^SiH_4_ Ref. [9]	^28^SiH_4_	^29^SiH_4_ Ref. [9]	^29^SiH_4_	^30^SiH_4_ Ref. [9]	^30^SiH_4_		(When Defined)
1	$1\;(1,0F_{1})$	0100E	$0001F_{2}$	−5.0694654 (44)	−5.15123 (60)	−5.06113221 (71)	−5.165 (12)	−5.053409 (20)	−5.0824 (92)		$-\left(\sqrt{3}\right)R_{24}+\left(\sqrt{3}/10\right)F_{24b}$
2	$2\;(2,0F_{2})$	0100E	$0001F_{2}$	−2.20982 (25)	−1.9509 (16)	−2.21014	−1.905 (26)	−2.210459 (64)	−2.125 (27)	×10^−2^	(undefined)
3	$3\;(1,0F_{1})$	0100E	$0001F_{2}$	−1.27456 (61)	−1.1996 (24)	−1.27513	−1.256 (42)	−1.275703 (47)	†	×10^−4^	$\left(3/4\right)R_{24}^{01}+\left(3/4\right)F_{24a}+\left(9/80\right)F_{24b}$

^†^ Fixed value. ^‡^ This factor applies to the six previous columns.

**Table 7:** Effective dipole moment parameter values for the $\nu_{2}$ and $\nu_{4}$ bands of all three silane isotopologues. Standard deviation is indicated in parentheses in the unit of last two digits.

Parameter	Order	Ω(K,nC)	$\left\{s\right\}\;\;C_{1}$	$\left\{s^{\prime }\right\}\;\;C_{2}$			Value/Debye				$f^{\;‡}$
$P_{1}-P_{0}$					^28^SiH_4_ Ref. [9]	^28^SiH_4_	^29^SiH_4_ Ref. [9]	^29^SiH_4_	^30^SiH_4_ Ref. [9]	^30^SiH_4_	
1	1	1 (1,$0F_{1}$)	$0000A_{1}$	0100E		2.768 (37)		†		†	×10^−4^
2	2	2 (2,$0F_{2}$)	$0000A_{1}$	0100E		−2.450 (31)		†		†	×10^−5^
3	0	0 (0,$0A_{1}$)	$0000A_{1}$	$0001F_{2}$	3.9099 (17)	4.03036 (86)	3.9410 (36)	4.0579 (26)	3.9763 (45)	4.0782 (28)	×10^−1^
4	1	1 (1,$0F_{1}$)	$0000A_{1}$	$0001F_{2}$	−3.955 (54)	−3.958 (34)	†	†	†	†	×10^−4^
5	2	2 (0,$0A_{1}$)	$0000A_{1}$	$0001F_{2}$	−3.074 (37)	−1.52 (66)	†	†	†	†	×10^−5^
6	2	2 (2,0E)	$0000A_{1}$	$0001F_{2}$		3.72 (26)		†		†	×10^−6^
7	2	2 (2,$0F_{2}$)	$0000A_{1}$	$0001F_{2}$		−5.78 (25)		†		†	×10^−6^
Data (intensities)						472		37		55	
*J*_max_						21		13		15	
*d*_rms_/%						3.116		4.464		3.601	
St. Dev.						3.813		4.081		2.867	

^†^ Fixed to ^28^SiH_4_ value. ^‡^ This factor applies to the six previous columns.

**Table 8:** Rotational–vibrational transitions of SiH_4_ in SiCaSDa. Polyad schemes are described by ($i_{1}$; $i_{2}$; …; $i_{N}$) multiplets as explained.

Transitions	Number Dipolar	Dipolar Wavenumber	Dipolar Intensity
		cm^−1^	cm^−1^/(molecule.cm^−2^)
^28^SiH_4_			
Scheme 1 (2, 1, 2, 1)			
$P_{1}-P_{0}$	6649	714–1154	1 × 10^−23^–6 × 10^−19^
^29^SiH_4_			
Scheme 1 (2, 1, 2, 1)			
$P_{1}-P_{0}$	6920	732–1180	1 × 10^−23^–6 × 10^−19^
^30^SiH_4_			
Scheme 1 (2, 1, 2, 1)			
$P_{1}-P_{0}$	6521	711–1154	1 × 10^−23^–6 × 10^−19^
Total	20,090		

## Data Availability

The data are freely available at the following address: https://vamdc.icb.cnrs.fr/PHP/SiH4.php, accessed on 1 June 2024.

## Abbreviations

The following abbreviations are used in this manuscript:

STDS	Spherical Top Data System
XTDS	Xtended Top Data System
RMS	Root Mean Square
